# A solidarity paradox – welfare state data in global health data economy

**DOI:** 10.1177/13634593211069320

**Published:** 2021-12-29

**Authors:** Karoliina Snell, Heta Tarkkala, Aaro Tupasela

**Affiliations:** University of Helsinki, Finland; University of Helsinki, Finland; University of Helsinki, Finland

**Keywords:** data economy, health data, solidarization, solidarity, welfare state

## Abstract

Nordic welfare states have well institutionalised practises of gathering health and social wellbeing data from their citizens. The establishment of population registers coincided with the building of welfare state institutions and a social contract relying on solidarity. During the last decade, the significance of Nordic registers and health data has increased and they have become sources of economic value. Recent policies expect registers, health data and biobanks to attract international investments, making Nordic countries world-leaders in the global health data economy. In this article we question the conditions and boundaries of solidarity in the emerging data-driven health economy. We argue that the logics of welfare state and data-driven health economy create a paradox – the data economy is not possible without the welfare state data regime, but the logic of data-driven health economy contradicts the value bases of the welfare state data regime and therefore the justifications for data gathering and use become questionable. We develop the concept of *solidarization* to describe the process by which individuals are expected to behave in a solidaristic way to support data gathering and related policy processes. We demonstrate the solidarity paradox through a recent legislative and data infrastructure reform in Finland and discuss it in relation to academic literature on solidarity.

## Introduction

State collected statistics and registers have been an integral part of health care, governance and intervention in the Nordic welfare states for decades (Desrosiéres, 1998). In the Nordic countries – Finland, Denmark, Sweden, Iceland and Norway – the establishment of data gathering practices, registers and infrastructures happened together with the construction of welfare state institutions in the 1960s and 1970s and was linked to the establishment of universal public health care and social services. More recently, however, the solidarity and reciprocity bound foundations have been connected to visions of a global data market, where state collected data and samples are seen as an asset whose value is derived from business, rather than public health and wellbeing alone (Tupasela, 2021). The Nordic welfare states have been moving towards developing broader frameworks to facilitate a more full-scale data sourcing and re-use strategy (Tarkkala et al., 2019; Tupasela et al., 2020; Cool, 2016; Holm and Ploug, 2017). In this article, we describe and analyse the changing regimes of gathering and using heath data, and the notion of solidarity in relation to these efforts. Our argument is that the justifications for gathering data are based on solidarity associated with the welfare state of last century, while the context and scope of data use has changed dramatically since then. We call this slodarity paradox.

Our analysis explores solidarity as a key value associated with the Nordic welfare states and how solidarity is compatible or incompatible in the current context of the global data-driven health economy, where the role of the state as data collector and extractor can be approached through the concept of competition state or commercial nationalism (see e.g. Jessop, 2002; Pelkonen, 2008; Volcic and Andrejevic, 2016). We argue that the logics of the welfare state and data-driven health economy create a paradox – being part of the data economy is dependent on the welfare state practices and solidarity that rests on reciprocal virtuous circles between the state and the citizens. At the same time, the logic of data-driven health economy dismantles the existing bases of solidarity since data extraction is focussed on the generation of private profit. This contradicts the justification of the welfare state data gathering, since a promise of profit itself is not necessarily enough to justify the uses of citizens’ personal data as a resource for economic activity and wealth outside data’s original, primary context (Snell 2019; Holm and Ploug, 2017).

Welfare states have been eager to support their economies throughout their histories, but in this new constellation population-based data would be a ‘direct input to capital’ (Couldry and Mejias, 2019a: 5). We argue that given that the promises of data economy are framed mainly as financial and appear vague and unpredictable, referring to solidarity helps to frame the new activities as morally justified. We therefore introduce the concept of *solidarization* to address how people are expected to be part of the data economy and continue to share an ethos of common responsibilities in a changing environment.

Our article provides new inputs to the discussions about solidarity (Hechter, 1988; Komter, 2005) in biomedicine (Chiapperino and Panese, 2020; Prainsack, 2018; Van Hoyweghen and Aarden, 2021) and data-driven societies (Hoeyer, 2016; Zuboff, 2015). Our aim is not to develop the concept of solidarity as such, but to show through the concept of solidarization how the idea of mutual obligations and benefits can be utilised to foster certain policies even when the actual policy outcomes are unclear and possibly detached from the premises the persuasion is built on.

We analyse this solidarity paradox and exemplify the argument with the help of a recent legislative reform in Finland. The reform is an effort to streamline the secondary use of health and welfare data, and to make data easily available for innovation, knowledge management and other purposes for both public and private users, nationally and internationally. We discuss the implications of this change in relation to the legitimacy of Nordic data regimes and the way people and their health citizenship with universal health care figure in it. These questions are not unique to the Nordic countries and our findings have relevance in many other contexts, such as the exploitation of NHS data in the UK by private companies. Many countries in the EU have made efforts to organise and pool together health data for wider uses (Vezyridis and Timmons, 2017; Wyatt et al., 2020). The EU, for example, is developing a common European Health Data Space (EHDS), which would allow for the movement of health data for treatment and research across borders (European Commission, 2019).

In the following two sections, we describe two different data regimes: the welfare state data regime and the regime of data-driven health economy. Then we present theoretical viewpoints to solidarity. After that, we exemplify the themes we have discussed through the recent legislative and institutional change in Finland. Finally, we draw theoretical conclusions about solidarization and the two data regimes.

## Welfare state data regime

Gathering information to population registers was routine in the Nordic countries already in the 18th century. The causes of death, for example, have been registered in Sweden since 1751. The centralised collection of systematic health and social wellbeing data and its use in research, as well as the provision of services has been taking place for more than half a century (see e.g. Bauer, 2014). The Danish cancer register was founded in 1943 and the Finnish cancer register in 1953. Electronic records related to health and illness similarly date back decades as well (Räisänen et al., 2013; Rosén, 2002). The establishment of these modern data gathering practices, registers and infrastructures coincides with the construction of welfare state institutions and universal health care and the need to identify individuals as subjects of welfare services (Alastalo, 2009). They are also part of a broader development of epidemiological research that was part of state-led efforts to improve public health surveillance. Each person needed to be identified accurately to ensure that obligations and rights were assigned correctly, while simultaneously producing statistics for governance (Redfern, 1989). Welfare state data collection – or what we call the *welfare state data regime* – also gave birth to the personal identification number, which is issued to every Nordic resident and utilised extensively in both public and private services (e.g. Alastalo and Helén, 2021; Bauer, 2014; Cool, 2016). In comparison to many other countries, the personal identification number of Nordic residents – and the legal structure and social practices associated with it – makes it possible to combine data from different registers relatively easily and without public controversy (Holm and Ploug, 2017). The PIN also allows for systematic public health surveillance of the entire population. This is a unique characteristic of the Nordic countries (Nordforsk, 2017).

Public health care and social services have been a core element of the Nordic welfare state model where citizens are entitled to universal health insurance and public medical services (Helén and Jauho, 2003). Data from the population registers, patient records and public health research has been used to advance national public health. In addition, the state received knowledge about its population and the need to allocate resources and services.

The welfare state data regime has been based on a social contract that relies on values such as reciprocity, universality, equality and solidarity. Nordic welfare states have been linked with ‘efficient organisation of society’ (Bauer, 2014: 204) and they have had a balancing and mediating relationship with the market economy in these societies (Kettunen, 2012; Stråth, 2004). This ethos of a shared understanding of obligations, entitlements and exchanges among the public authorities, society and the individuals is still a crucial framework in which people consider issues related to medicine, and their personal contribution to medical care and research (Lipworth et al., 2011). The support for the Finnish welfare state among Finnish residents is strong and there are few signs of decreasing public support for welfare policies (Muuri et al., 2012), even when the welfare states have also faced criticism and have been called to renew themselves (e.g. Hilson, 2008; Kettunen, 2012; Stråth, 2004).

## Data as the source of economic value

The welfare states are struggling with an ageing population, growing health care costs and are trying to find sustainable ways to fund services (Bengtsson, 2010; Thijssen, 2016). During the last decade the significance of Nordic registers, biobanks and health data has been elevated to a new level. Population registers have been dubbed as the ‘Nordic goldmine’ (Tupasela et al., 2020; Kongsholm et al., 2018). High expectations are placed on human biological samples and population data – such as register, genomic and health data – to generate economic growth and improve people’s health. Harnessing the potential is seen as a precondition for economic growth, sustaining of welfare services and improvement of personal and population health (Tarkkala et al., 2019).

Within this socio-technical vision, innovations and businesses emerge and develop to produce wealth and services. This vision locates the data flows in new ways – previously they were situated and utilised in the spheres of state administration, governance, and services. In the new context, more and more resources are appropriated for the benefit of a business model where extraction and management of data is done in the name of economic profit. Instead of social insurance or service provision, the data is imagined as a profit generating resource, ‘“just there” for exploitation’ (Couldry and Mejias, 2019a: 5) for the purposes of business, innovation and politics (Tarkkala et al., 2019; Couldry and Yu, 2018 ). This trend towards the assetization of public health records and data can be seen in several public healthcare systems, such as the Britain’s NHS (Hawkes, 2016; Vezyridis and Timmons, 2021).

The effective utilisation of data has become one of the central goals of health and innovation policies, in the Nordic countries (e.g. Tupasela et al. 2020; Tarkkala et al. 2019; Hoeyer, 2016) and national strategies all over the globe and multinational policies aim to capture the potential of health data (see Bell, 2017; Ministry of Health, 2017; Ministry of Social Affairs and Health, 2015). Moreover, health data business has been identified as one of the biggest growing branches, both in relation to development of technology, and the circulation of money. Global companies, such as Google and Amazon are making inroads to the health care data market (Sharon, 2016; Wingfield et al., 2018). Data-driven economies are built on data-intensive practices, which implies the need to gather more and different types of data on more people with unprecedented utilisation possibilities (Hoeyer, 2016; Hogle, 2016; König, 2017).

Herein we identify a turn from welfare state data regime towards the *regime of data-driven health economy.* This turn represents a movement towards a competition state (e.g. Jessop, 2002; Pelkonen, 2008) with emphasis on competitive advantages, as well as economic growth. Thus, the development to capitalise and utilise data depositories in new ways can be seen in the wider context of how ‘old welfare-state institutions have been modified to serve new competition-state functions’ (Kettunen, 2012: 22). However, with data-driven health economy, it is unclear what obligations and rights individuals maintain in relation to their own data that is in public registries, biobanks and health records. The new data economies treat individuals as sources of data for both welfare services and data intensive businesses. We need to ask, what forms of reciprocity and solidarity does the data economy produce and entail? It is here that we recognise an area where earlier premises of solidarity shift into a new context with an ill fit. Competitiveness has been portrayed as incompatible with reforming the welfare state (Kettunen, 2019), while simultaneously it has been the welfare state that created the data – the competitive factor – as part of its operations (Tarkkala, 2019). In this sense, citizens become obliged to remain as endless sources of data to bolster national competitiveness.

At the same time, it is unclear how the benefits of that competitiveness return as concrete paybacks to public health care or the citizens. This departs from the more traditional concern the state has had of its population as a workforce. This post-Fordist perspective develops new ways in which labour is folded into systems of production (Couldry and Mejias, 2019a, 2019b; Zuboff, 2015), or a different version of labour altogether when being a mere accumulating data point – whether sick and diseased or healthy – potentially generates surplus in a data economy (see also Wienroth et al., 2019; Wyatt et al., 2020). This is by no means unique to Finland. For example, in the UK, NHS patients have been encouraged to participate in clinical research. According to Wienroth et al. (2019: 1457) this means that ‘patients have become assets to, not only recipients of, the UK national health care system’. Thus, expectation of revenue and surplus is in this sense to be found in universal health care systems.

Some of the Nordic countries, tend to underline more the possibilities to support the welfare state through investing in health data infrastructures and the role of the public sector (Jensen and Svendsen, 2021) while others, such as Finland, lean heavily on national competitiveness and possibilities to involve private companies and attract international business to utilise the data. The data no longer serves the welfare state and its services alone. Instead, value is actualised through the circulation of data within private businesses, which seek to refine it into products and services in the global economy. Those who benefit might indeed be the citizens of the welfare state, but this happens through economic growth and market mechanisms instead of the more direct manner associated with welfare state data regime (e.g. public health services). The welfare state data regime is however required for the massive data gathering. Rose described the Icelandic welfare state context for genetics, already before the current turn to data economies:

*Potentially immense innovation, actively pursued by global pharmaceutical companies and venture capital, requires as its precondition a universal health care system. Only the old welfare states have universal health care records. Not for the first time does the relationship between the organisational structures of health care provision and the development of genetics come into visibility and importance. For pharmacogenomics, only the old welfare states offer what they speak of in their depoliticised language as a ‘good population’*. (Rose, 2003: 77)

Unlike private companies, welfare states maintain ‘complete’ and socio-economically broad records of their populations. While companies like Google, Amazon and Facebook have massive amounts of big data that they collect from their users continuously; customers are not obliged to use their services comprehensively, thus creating dis-continuities and holes in their data sets. Although, these data sets may be useful for marketing and predictive analytics, when it comes to pharmaceutical and health research, they come with biases and blind spots. Within this context, welfare states are seen as a ‘gold mine’ for complete data that date back in history and come with follow-up information on disease outcomes and mortality.

## Theorising solidarity

According to Bergmark (2000), solidarity is one of the main values linked to the Nordic welfare state model. Also at the European level, there is an understanding that healthcare systems should be based on solidarity, which guides the design of the system and allocation of resources (Prainsack and Buyx, 2017; Houtepen and Ter Meulen, 2000). Furthermore, other values, such as equality and universality are associated with welfare states. We focus on solidarity, since the concept has gained popularity in recent years (e.g. Ahola-Launonen, 2019). As Buyx and Prainsack (2018: 537) put it, ‘Solidarity is en vogue’ in bioethical discussions. *First*, within the academic literature concerning health research, biobanks and other health data infrastructures (e.g. Hendrickx and Van Hoyweghen, 2018; Prainsack and Buyx, 2017; Woods, 2016). *Second*, genetic knowledge has been seen to connect people in new ways – creating biosociality and solidarity between people with shared genetic heritage (Rabinow, 1996; Strathern, 2005). *Third*, solidarity is used in policy contexts as a rhetorical tool to convince people to participate in biobanks and health data projects (Snell, 2019; Council of Europe, 2006; European Commission, 2005).

Solidarity is used as a descriptive term to analyse principles of participation in and governance of health data systems, as well as a normative term to justify health data projects and push people to choose a particular course of action (Gould, 2018; Peeters, 2013). The notion of solidarity represents a type of politics of values, which is used to justify policy and legal implementations (Snell, 2019). The solidarity approach complements and, in some cases, replaces other normative arguments, which have previously relied on improving efficiency (Tarkkala, 2019) or emphasising the commercial expectations associated particularly with genomes research (Tarkkala et al., 2019). Although communitarianism maintains many similarities to solidarity and has a strong grounding in sociological discussion, we prefer to use the term solidarity instead since terminologically it better reflects the current discussion related to health data and biobanks (cf. Prainsack and Buyx, 2011).

There are many theoretical viewpoints to solidarity. Moral philosophy, bioethics, sociology and welfare state research tend to highlight different aspects and origins of the concept: religion, labour movement, French revolution or German philosophy, for example (Houtepen and Ter Meulen, 2000; Kelliher, 2018). In most definitions, however, solidarity is linked to making sacrifices or giving support to members of a group or people one recognises similar in some way (Scholz, 2019; Trampusch, 2007). Peoples’ mutual acknowledgement of something they share is an important element in justifying and enabling the kind of thinking also social contract theories have promoted (Prainsack and Buyx, 2011: 7). In their book on biomedicine and solidarity, Prainsack and Buyx (2017: 43) define solidarity as ‘enacted commitments to accept costs to assist others with whom a person or persons recognise similarity in a relevant respect’. We want to focus the attention to some specific aspects of solidarity. *First*, we point to the nationalistic character of solidarity in the welfare states. *Second*, we focus on the latter part of the definition of solidarity: the scale and quality of group membership or perceived similarity – that is to whom actions or support are directed. *Third*, we discuss the temporal agency of solidaristic action or enacted commitments.

The concept of solidarity has been traditionally used in connection to nation states. In the welfare state ideology the support and costs are shared mainly through taxes and allocated within the state, leading to a type of solidarity that Scholz (2015) has called civic solidarity. Solidarity in the welfare state has also been associated with nationalism and homogeneity of the population (Hilson, 2008; Keskinen et al., 2019; Kettunen et al., 2015). Some authors have underlined how referring to nations, excludes other people from the sphere of solidarity (Gunson, 2009). There have been calls for a more inclusive notion of solidarity that could extend the ideas of group membership to transnational contexts (Kelliher, 2018). Gould (2018), for example, argues for overcoming the notion of unitary solidarity that is manifested among citizens of one nation to networking solidarity that has a more transnational character. She aims to reconceptualise solidarity relations as ‘contributing to the emergence of more democratic forms of transnational interaction within regional or more fully global frameworks of human rights’ (Gould, 2007, 148). According to her, in networking solidarity, people will form networks and temporary groups that are dedicated to specific causes, such as supporting people effected by natural disasters. People or associations across nations take joint efforts to eliminate suffering or overcome oppression in a project-oriented manner. Gould (2007) also maintains that understanding solidarity as linked to human rights rather that citizenship can contribute to the emergence of more democratic forms of transnational interaction. In this respect, the group membership and similarity required by solidaristic action would extend national boundaries to cover all humankind.

A temporal viewpoint to solidarity comes from Peeters (2013), who has examined the rhetorical use of responsibility and solidarity in modern welfare states. According to him, there is a notable shift in the usage of the terms. Formerly, solidarity and responsibility were used to justify state intervention as individuals could rely on solidarity if they fell victim to externalities, such as unemployment and illness. In this ‘European’ solidarity paradigm, the state’s role has been to facilitate wellbeing and protect citizens from harm and abuse (Scholz, 2015). Peeters (2013) argues that in the contemporary welfare state the concepts of solidarity and responsibility are turned into arguments for citizens – that they should evaluate the consequences of their behaviour before they make choices and act. He claims that this demonstrates a temporal shift from ex post to ex ante responsibility which forms the logic behind the governance strategies of the new welfare state. Where the concepts ‘appeal to the liberal values of individual autonomy but are also used to justify a specific type of state intervention designed to incentivise, entice, persuade and nudge citizens to choose for a specific course of action’ (Peeters, 2013: 593).

This points to a solidarity that emphasises the individual actions that need to be of a certain type to promote solidarity, instead of common social action that is targetted to improve the health or social situation of people in deprived life conditions. Solidarity in this sense implies taking responsibility not only of one’s own health but also of producing public value. While Peeters discusses both solidarity and responsibility, he focuses on the idea of responsibilisation. Responsibilisation of citizens and patients has been much discussed in academic literature (e.g. Ahola-Launonen, 2019; Trnka and Trundle, 2014; Viens, 2019). There has been less discussion about *solidarization* of individuals – how citizens must take particular types of action or are expected to behave in a particular manner in the name of solidarity, in order to demonstrate willingness to further and promote policy goals. Solidarization is thus a phenomenon of governance and persuasion. Similarly, Börner et al. (2020) have argued that autonomy is no longer an individual aspiration but a social obligation. In the following section we will develop some of these aspects of solidarity by reflecting the example of secondary use of health and social data in Finland.

## Finnish reform of secondary use of health and social welfare data

Similar to Denmark in fostering personalised medicine, Finland too, has sought to strengthen the role of the state in terms of organisation and control of access to data (see Jensen and Svendsen, 2021; Terkildsen et al., 2020). In 2015, the Finnish Ministry of Social Affairs and Health set a working group to renew legislation concerning the secondary use of health and social welfare data. Despite the persistent rhetoric of ‘uniqueness of Finnish data’ and ‘Nordic goldmine’ (Tupasela, 2021; Tarkkala, 2019; Tarkkala and Tupasela, 2018), the existing legal framework for secondary uses of data was seen as outdated and insufficient. The purpose of the renewal was to create a single permit authority and make the combination of different data resources easier. The Finnish data resources are dispersed in several information systems and managed by different register keepers such as National Institute of Health (THL), the Social Insurance Institution of Finland (Kela), the Population Register Centre, Statistics Finland, and regional state administrative agencies (Findata, 2020). The reform aimed at streamlining the slow and laborious processing of data requests from different authorities and speeding the access to data by creating a one-stop-shop that also combines the data (Aula, 2019; Sinipuro, 2017). The amount of data that is available is vast and includes, for example, medical records and prescriptions, registers of child welfare, cancer, work and earnings, family relations, mass screening, abortions, infectious diseases, as well as various other registers of health and social care (Neittaanmäki et al., 2018).

In addition, the goal was to make data accessible for more uses than before, thus covering not only research and statistics, but also development and innovation, teaching, knowledge management, monitoring, steering and official planning. This viewpoint drew its reasoning from the argument that not using data sources to their full extent could be considered *unethical* (Jones et al., 2017; see also Prainsack, 2015) and economically inefficient. The renewal process was part of the national ‘Health Sector Growth Strategy’ (Ministry of Economic Affairs and Employment, 2014).

The Finnish government passed the proposal in 2019 and Findata, the national Health and Social Data Permit Authority and digital operator, was established at the beginning of 2020. The legislative and infrastructural reform took place at the same time as other major initiatives in the health data field: the planning of a national genome centre and drafting of genome legislation, as well as strengthening coordination between biobanks and the renewal of the biobank law. Notions such as ‘joint national project’ have been used to describe these efforts and to mobilise stakeholders (Snell, 2020). Since the Health Sector Growth Strategy formed the backdrop for all these projects, it is understandable that these reforms have had a strong economic emphasis. Health and wellbeing are increasingly concerns of economic potential and growth and data sharing becomes the *starting point* for advancing health (Tarkkala et al., 2019; Couldry and Yu, 2018).

In this new context, individuals are placed in a position, where they are nudged towards having an ethical responsibility to contribute to common good by caring for themselves and sharing data (Couldry and Yu, 2018). However, in the Finnish context, providing data for the common good is not mostly a question of a solidaristic choice. All the registers and databases included under the jurisdiction of Findata are composed of data gathered on legal bases. This means that the data is gathered automatically, and Finns are not asked for consent for the original or secondary use of data. In most cases people are not even aware that their data is being collected and processed (Snell and Tarkkala, 2019). Many definitions of solidarity emphasise that solidarity must be expressed through action or that it is an enacted practice (Gunson, 2009; Prainsack and Buyx, 2017), which is not evident in this case. We agree with Prainsack and Buyx (2017: 103) who claim that ‘data collection that people have not agreed to, or are not even aware of, cannot be seen as part of a solidaristic practice. This is because solidaristic practice always includes intentionality and decision-making’.

This sort of obligation or top-down solidarity mandated by regulation or law (Woods, 2016; Yeh, 2020) has been identified to be part of welfare state health regime (Helén and Jauho, 2003), but as we argue, this relationship is now taking place in a context that is swiftly developing towards a ‘global data economy’. While the data utilised in the name of economic growth is still based on state collected records for administrative purposes, the scope and justifications of data use have changed. A similar contextual change has been identified also in the UK, where ‘by virtue of being an NHS patient, of being able to use NHS services, patients and publics also become part of the research system’ (Wienroth et al., 2019: 1456). This research system, much like the Finnish case, would be based on universal health care system, the data collected in the routines of NHS, and it indicates a change in the relationship between the health care providers and patients (see also Sterckx et al., 2016). Wyatt et al. (2020: 2) describe this change being about translating the patients of NHS ‘into a digital, searchable and knowable population for the needs of the research community and, by extension, the UK economy’. The Finnish case moves beyond the clinical setting by underlining the secondary uses of multiple kinds of publicly collected health and social data through new legislation.

## Solidarity in global health data economy?

In this section, we return to the three aspects of solidarity we introduced earlier to demonstrate our argument about a solidarity paradox.

First, argumentation supporting secondary use of data points to a national continuum – to a national project, competitiveness and national solidarity. Data gathering and utilisation is described as business as usual. It is presented that there is nothing new in data collection and use – registers have existed for many decades and hospital records, for example, have been used for research before. This argumentation is in line with the nationalistic tradition of solidarity. Everyone is expected to contribute to the common project, as they have done before (Snell, 2019). The focus on previous justifications for data gathering and use downplays the recent changes in the context: possibilities to combine, analyse and distribute data in novel ways for both public and commercial interests, inside and outside the nation state.

Focussing on the national continuum frames Finnish citizens as the beneficiaries of data economy. However, it is unclear what services and benefits emerge from being sourced for data – there is no vision about how the state would redistribute data-based wealth. Although policies and private sector lobbying make promises of a brighter future with better services, the concrete examples of results remain far and few. As the context of data-driven health economy is global and data is opened for wide future uses, the recipients of new services, medicine or knowledge are not clearly defined and can be located anywhere in time and space.

This brings us to the second point. Data-driven health economy presupposes global, universal and transnational solidarity focussing on humanity rather than nation state bounded solidarity. However, the focussing on national competitiveness and continuum downplays aspects of global, universal or transnational solidarity. Thinking about solidarity as universal, provides another problem in relation to global health data economy. It is not at all clear that the whole humanity will benefit. A common concern among citizens (Snell, 2018), as well as bioethicists (Gould, 2007; Hayden, 2007; Knoppers, 2000) is that only relatively few will benefit and sound principles for benefit-sharing are needed. Structural injustices, globally as well as locally, place only some illnesses, nationalities and groups as research subjects, targets of health intervention or winners. It is difficult to establish international groups or networks that support a cause – network solidarity Gould (2018) is arguing for – because the systems aim at making data available for many and unspecified purposes. Most definitions of solidarity rest on acknowledging certain similarity with the people who are vulnerable or in need of solidarity. Solidarity thus requires certain amount of perceived likeness, a shared, common ground. The problem with the developments in large data projects and secondary use of data is that the groups or people possibly benefitting cannot be defined. Secondary uses of health data can be used to develop services and conduct research that is not yet anticipated, but solidarity must be enacted now.

This leads to the third aspect we want to highlight, which is the temporal and agential shift in solidarity. According to Peeters (2013), before, the state’s interventions happened after an individual got sick or unemployed, and now the idea is for the citizens to act beforehand. People are expected to behave in preventive and anticipatory ways, and free-riding or selfishness are regarded as hindrances to the shared ambition and solidarity (Snell, 2019). This is what we term solidarization. People are also expected to accept data gathering and utilisation as part of their moral citizenship. The process of solidarization in Finland has been incremental, taking place over two decades and relating to changes in several legislative frameworks. At the same time, however, in relation to innovation policies and state competitiveness, solidarization has been developed systematically to fit the needs of policymakers and the research community.

## Conclusions

We have identified a shift of regimes for collecting and utilising population data and argued that data-driven health economy requires the welfare state data regime and solidarity to function, but it contradicts the solidaristic value bases of the welfare state, which has enabled the data extraction and existence of the registers and databases. This is what we term as solidarity paradox. We argued that contemporary health data utilisation is part of global economy, and it departs from the national, public health thinking of the welfare state data regime moving the data registers and depositories towards the context of competition states. Unlike traditional notions of solidarity, the uses of data are undefined and future-oriented. This makes it difficult for people to identify the beneficiaries of health data economy, or who would be the vulnerable people that they express solidarity to.

Solidarity is often seen as a solution to the tension between personal decisions and common good. For example, Prainsack (2018: 36) has argued that:

*Solidarity-based frameworks start with what people need, want, and do, and, on the basis of this, seek to create the circumstances within which practices that support others can be more easily ‘scaled up’ to become more widespread. Because of its initial grounding in actual practices and values, solidarity is less vulnerable than other pro-social concepts to being used to justify pushing people into doing something they do not want to do for the sake of collective benefit.*

Our analysis contradicts this statement and shows that solidarity is used in connection to old practices that do not account for the changing context of data use. Therefore, solidarity seems to be another concept that tries to motivate people to act as wanted (nudge) by those promoting global health data economy. We have termed this process *solidarization*, whereby people are expected to behave in a certain way with regards to the data that is collected from them. In relation to the Finnish health and social welfare data, this is taken even further. Finns cannot consent for data gathering or opt-out from most parts of the data infrastructure. Thus solidarity is presumed or imposed without the knowledge or agency of people. Public health care system and welfare state registers are turned into assets in the global health data markets, which might contrast with the way people have used to think about their health care and data gathered through public services.

The virtuous circles these are expected to create are promissory by nature. They lack concrete situatedness in the everyday life, as well as clarity in terms of how they figure and reconfigure the relationships between the state and the citizens (see also Wyatt et al., 2020). The visions and beneficiaries of data economies tend to be global rather than national, general rather than concrete and economic rather than health-related. In the new situation, the values linked to the universal health care system might not travel easily into more commercial and complex context of innovations, data driven medicine, building of public-private partnerships and making business out of clinical care. If it is not clear who gets the benefits and in what form, the acceptance to data and sample collecting by the state might turn to be problematic (Snell and Tarkkala, 2019). Especially if there seems to be no transparency or collective oversight about the way health data serves its users in its many contexts (see also Prainsack, 2017).

Couldry and Yu (2018) claim that in the global data flows and markets the crucial question whether data should be collected at all remains undiscussed. Instead, the discussions are often about the uses of data. In the context of our case, both these questions are entangled: should data be collected at all for these open-ended purposes? As long as there is no discussion about potential negative outcomes of these developments to the people whose data is being appropriated and used, the promises seem to remain as legitimising discourse about unprecedented good(s) that follow (see also Couldry and Yu, 2018). Thus, people are expected to approve and become solidarized to the new data practices based on promises, persuasion and expectations, without thorough discussion about the contexts of new data uses and the respective values of these practices. An important question is, will solidarization result in conditional citizenship – only those who adjust to solidarization will be entitled to services.
